# Combining Topic Modeling, Sentiment Analysis, and Corpus Linguistics to Analyze Unstructured Web-Based Patient Experience Data: Case Study of Modafinil Experiences

**DOI:** 10.2196/54321

**Published:** 2024-12-11

**Authors:** Julia Walsh, Jonathan Cave, Frances Griffiths

**Affiliations:** 1 Warwick Medical School University of Warwick Coventry United Kingdom; 2 Department of Economics University of Warwick Coventry United Kingdom; 3 Centre for Health Policy University of the Witwatersrand Johannesburg South Africa

**Keywords:** unstructured text, natural language processing, NLP, topic modeling, sentiment analysis, corpus linguistics, social media data, patient experience, unsupervised, modafinil

## Abstract

**Background:**

Patient experience data from social media offer patient-centered perspectives on disease, treatments, and health service delivery. Current guidelines typically rely on systematic reviews, while qualitative health studies are often seen as anecdotal and nongeneralizable. This study explores combining personal health experiences from multiple sources to create generalizable evidence.

**Objective:**

The study aims to (1) investigate how combining unsupervised natural language processing (NLP) and corpus linguistics can explore patient perspectives from a large unstructured dataset of modafinil experiences, (2) compare findings with Cochrane meta-analyses on modafinil’s effectiveness, and (3) develop a methodology for analyzing such data.

**Methods:**

Using 69,022 posts from 790 sources, we used a variety of NLP and corpus techniques to analyze the data, including data cleaning techniques to maximize post context, Python for NLP techniques, and Sketch Engine for linguistic analysis. We used multiple topic mining approaches, such as latent Dirichlet allocation, nonnegative matrix factorization, and word-embedding methods. Sentiment analysis used TextBlob and Valence Aware Dictionary and Sentiment Reasoner, while corpus methods including collocation, concordance, and n-gram generation. Previous work had mapped topic mining to themes, such as health conditions, reasons for taking modafinil, symptom impacts, dosage, side effects, effectiveness, and treatment comparisons.

**Results:**

Key findings of the study included modafinil use across 166 health conditions, most frequently narcolepsy, multiple sclerosis, attention-deficit disorder, anxiety, sleep apnea, depression, bipolar disorder, chronic fatigue syndrome, fibromyalgia, and chronic disease. Word-embedding topic modeling mapped 70% of posts to predefined themes, while sentiment analysis revealed 65% positive responses, 6% neutral responses, and 28% negative responses. Notably, the perceived effectiveness of modafinil for various conditions strongly contrasts with the findings of existing randomized controlled trials and systematic reviews, which conclude insufficient or low-quality evidence of effectiveness.

**Conclusions:**

This study demonstrated the value of combining NLP with linguistic techniques for analyzing large unstructured text datasets. Despite varying opinions, findings were methodologically consistent and challenged existing clinical evidence. This suggests that patient-generated data could potentially provide valuable insights into treatment outcomes, potentially improving clinical understanding and patient care.

## Introduction

### Background

Spontaneously generated online patient experience (SGOPE) data collected from social media platforms are a rich data source for natural language processing (NLP) tasks [1-4]. Providing patient-centered perspectives [5,6] on the posters’ experiences of disease, treatments, and health service delivery rather than the researcher-driven focus of published literature [7], SGOPE data are increasingly recognized as having the potential to transform clinical care and research [6,8-14].

Current estimates suggest that 3.6 billion people worldwide are currently using social media, with numbers forecast to increase to 4.4 billion by 2025 [15]. Social media were originally seen as being mostly used by younger people, but a 2019 US study showed that 73% of individuals aged 50 to 64 years and 45% of those aged ≥65 years used at least 1 form of social media [16]. SGOPE is recognized as being able to include a wider range of demographic groups, including many who may previously have been seen as “hard to reach” [17-19].

Modafinil is an oral wakefulness-promoting drug originally developed in the 1990s that is licensed by the UK National Health Service purely for narcolepsy, although its Food and Drug Administration classification in the United States allows it to be prescribed “off-label” for a wide variety of conditions [20]. Modafinil targets symptoms of fatigue seen in many clinical presentations; however, current randomized controlled trial (RCT)–based evidence regarding its efficacy for treating other conditions is inconclusive [21]. Having acquired a reputation as a “study drug,” modafinil has sparked a large volume of online discussion about posters’ experiences of taking it for both therapeutic and enhancement purposes.

Patient narrative is already recognized as a tool that can help patients, clinicians, and researchers [22,23]. Containing a mix of both objective and subjective views, SGOPE data provide a unique perspective on the way that patients perceive, manage, and react to their conditions, as well as how such conditions impact their life, their treatments, or other aspects of their health [24].

Although evidence-based medicine has been defined as the integration of the best research evidence with real-world clinical expertise and patient values (Sackett et al [25]), in reality, the pyramid-shaped hierarchy of evidence quality ensures that it is the findings from RCTs and subsequent systematic reviews, rather than any other form of knowledge, that tend to dominate and be reflected in the clinical guidelines [26-28].

The need for a plurality of evidence-generating methods is already recognized [29-31]. SGOPE represents a type of data that fall under the umbrella terms of real-world data (RWD) and real-world evidence. RWD include health care data generated from sources other than conventional RCTs, while real-world evidence is defined as evidence derived from the aggregation and analysis of RWD [32] and is argued to have significant advantages that can be used to supplement or augment RCT findings, including the ability to identify “clinical gaps” [33], indicating the effectiveness of an intervention in the real world, on much larger populations, and much faster than can be achieved within the artificial and highly constrained confines of an RCT [34,35]. Combining data sources such as SGOPE with new methods of analyzing unstructured data will enable the development of new and different approaches to knowledge and evidence generation.

Our previous study compared a thematic qualitative analysis with an NLP-based analysis of a small number of posts related to the therapeutic use of modafinil [21]. Eight main themes were identified from the posts, including details of the reasons for taking modafinil, conditions or symptoms, dosage, side effects, effectiveness, and outcomes in terms of quality of life, as well as details of other interventions whether previously tried, used concurrently, or subsequently moved on to. In this paper, we scale up this approach, using a combination of NLP and linguistic techniques to analyze a much larger dataset of modafinil experiences from a wide variety of social media platforms. We also compare the findings from some of the NLP tools used for the analysis to help future analysis of this type of data for health research.

### Methodology

NLP approaches can be divided into 2 main types: supervised, which requires large quantities of the data to be labeled with the features of interest; and unsupervised, which uses clustering techniques that allow the data to tell their own story. Despite the development of ever-larger language models, such as GPT-3, which can be extremely resource heavy [36,37], there is an argument that to try to move nearer to the ultimate goal of natural language understanding, which is required to understand the complexity of patient experiences, entails stepping back toward combining unsupervised, rules-based methods with those from corpus linguistics [38,39]. To replicate the inductive data–driven approach of qualitative studies, but on a much larger scale, this study uses unsupervised methods. These include varied methods of topic modeling, sentiment analysis, and linguistic analysis.

Whichever approach is selected, cleaning the data is one of the most important and time-consuming components of the study. The cleaning process is specific to each project—each dataset has its own characteristics, and each project requires specific features from the dataset to answer the research question—but it is important to try to maximize the quality of the processed dataset for each subtask; for instance, in topic modeling, the aim of preprocessing is to reduce noise and incoherence from the data [40], allowing the themes to emerge. Stemming and lemmatizing words to their root form enables this, whereas when assessing effectiveness, it is important to retain all relevant details to understand the nuanced context within the text. Taking too blunt an approach can result in the loss of potentially useful data.

Particularly suitable for exploratory and descriptive analysis, topic modeling can be used as a method for determining what people are talking about in social media by looking for underlying structure within the text [41]. Combining an inductive approach with quantitative measurement, topic modeling is a useful method for obtaining an insight into the concepts that are contained within documents in a similar manner to grounded theory [42], although it is not yet widely used in clinical NLP [43].

Sentiment analysis is a well-known and widely used technique within NLP that analyzes text for positive, neutral, or negative sentiment or emotion, aiming to extract an understanding of the meaning, mood, context, and intent. It has already been shown to be capable of reasonable agreement with online comments, including those rated using a Likert scale [44].

Causation is central to health care, both in understanding the onset of diseases or symptoms and the effectiveness of interventions or management strategies used to treat them [45]. Showing causation in health care using non-RCT data has been viewed as problematic. At both structural and cultural levels, causation is generally seen as something that can only be shown in empirical settings such as RCTs, where all confounding factors are controlled for, and the Humean principle of “same cause, same effect” can be repeatedly shown [46,47].

Causal dispositionalism is an alternative approach to causation, which may be relevant to this type of data. This takes a more nuanced view of how the characteristics or dispositions of both the intervention and the individual combine in complex ways to affect the effectiveness [48]. It suggests that population-level health research should be only 1 part of the evidence-generation process, and that it is listening to the patient narrative that can be the key to understanding their individual health needs [47]. One of the strengths of narrative data, such as SGOPE data, is that they enable both author and reader to make sense of the interplay of actions and contexts in the text in a way that conveys perceived causality [22]. The mantra “correlation does not equal causation” is justifiably used, but that leaves the question of how it is possible to determine causation.

Causation can be defined as a reaction between 2 events: a cause event and its consequence. The cause must precede the consequence and is counterfactual in that the consequence would not have occurred without the cause. While this sounds quite logical and straightforward, causation theories are not necessarily definitive explanations of how events occur but rather represent how humans make sense of, and understand, the world [49]. Williamson [50] argues that causation can be shown by identifying or understanding the underlying mechanism between a correlated cause and effect.

NLP methods still struggle with identifying potential causality; therefore, we used linguistic analysis to aid in this process. The language used to describe cause and effect can be crucial to understanding the semantic meaning of a text but is not always easy to identify. One method involves using transition words that link a reason to a consequence or indicate a sequence of events (Textbox 1).

**Examples of text that indicate sequential events.**

**Transition words**

- Firstly
- to begin with
- next
- then following this
- at this time
- now
- at this point
- previously
- before this
- after
- afterward
- subsequently
- finally
- at last
- simultaneously
- meanwhile

Traditionally, findings from health-based qualitative studies have been seen as anecdotal, unrepresentative, and not generalizable across populations [51]. This study examines how we can move toward combining personal evidence of a health effect from sufficient numbers of people to the point where it could be generalized and added to existing population-level evidence [47].

The aim of this study was to assess what can be learnt from an NLP-based analysis of a large quantity of unstructured SGOPE data. This can be broken down into 5 subquestions:

- To assess whether topic mining can elicit the themes that are contained in the data
- To explore how sentiment analysis can be used to assess perceived effectiveness
- To compare various methods of theme and effectiveness identification
- To assess whether linguistic analysis can identify perceived causality from the text
- To establish whether these techniques can be used to develop a methodology for this type of analysis

## Methods

### Overview

The dataset contained 69,022 publicly available social media posts and threads that included the terms *modafinil*, *provigil*, *armodafinil*, or *nuvigil* as of July 2017. The dataset was supplied by Treato Ltd, which was a web-based social media data mining service that collected publicly available health-related posts (ie, posts viewable by anyone without requiring log-in) from >10,000 global blogs and online forums. The company agreed to supply English-language data relating to modafinil use, using its own proprietary algorithms based on the Unified Medical Language System to create a searchable dataset that can be analyzed in aggregate [52].

Analysis code was developed using Python (version 3.8.5; Python Software Foundation) [53] in JupyterLab (version 3.0.15; Project Jupyter) [54]. Bearing in mind the need to retain as much context to the data as possible, as described in the Methodology subsection, we took a staged approach to data cleaning, initially performing a minimal level of transformation and parsing of fields. The time stamp field, originally formatted as *2011-01-01 00:00:00 UTC*, was simplified to *PostYear* to represent the year the post was published. Line breaks, paragraph breaks, and other extra spaces were removed. The URL field was parsed to identify the main website or forum name. New fields were created for subsite names. Having extracted the site name, it became obvious that many of the URLs contained either the name of the condition that was of primary interest to the poster or the title of the thread or question that they were referring to. Using clustering techniques, we were able to group and extract this detail from the URL. Three new fields were created to represent the second-level domain name, the site’s focus condition (if applicable), and the extracted thread titles. To maximize the options for analysis, the cleaned data were structured to include 3 additional fields: *TextOnly* (response only), *Title* (thread title), and *TextWithTitle* (thread title preceding each response). All references to dosage amount in *mg* were standardized to *xxxmg*. Exact duplicate posts and obvious spam posts were removed. After data deduplication and spam removal, all forms of author identification were removed. The restructured file was saved in CSV format for the next stages. The *TextOnly* and *Title* fields were exported as 2 separate corpora text files for linguistic analysis. Keeping them distinct avoided the possibility of the repetition of the title words skewing any frequency-based analysis. These steps enabled us to obtain a dataset that retained an optimal level of quality and flexibility and upon which further preprocessing could be performed specific to the individual task.

### Topic Modeling to Identify Themes

Topic modeling was the main method for theme detection. On the basis of a previous study that evaluated 4 of the most widely used bag-of-words topic modeling methods [55], we selected latent Dirichlet allocation (LDA) and nonnegative matrix factorization (NMF) for comparison because they were seen to deliver the most meaningful extracted topics. Both LDA and NMF use the bag-of-words approach, which disregards any order within the corpus and uses word frequency to generate topics. Although the LDA method has been the most widely used method for patient experience feedback [56], a previous study found that NMF yields better results than LDA when used for short texts [57]. Other comparisons between the 2 methods found that LDA output was more semantically interpretable with more distinct categories [58], while NMF was faster and therefore less resource intensive [59]. However, another comparison found the opposite [60]. Yet another study suggested that NMF returned higher quality topics than LDA on smaller datasets [61]. As part of the project involves identifying a methodology for this type of data that can be developed for use on other datasets, we compared the findings of both methods using the *gensim* (version 3.8.3) [62] and *sklearn* (version 0.23.1) [63] libraries as they relate to SGOPE data. Another package—Top2Vec (version 1.0.24) [64]—using word-embedding methods was released during the study and was included for comparison. Word-embedding methods work by considering each word in the context of its neighbors, creating a numeric vector where words with similar meanings are grouped together, which has been seen as a significant advance in trying to establish the meaning or topics of posts [65].

Additional preprocessing for the LDA and NMF methods included removing stop words and punctuation and converting all text to lowercase. The stop word list was extended to include common name variations for modafinil. Bigrams and trigrams were generated; text tokens were lemmatized; and part-of-speech (POS) tags relating to nouns, adjectives, verbs, and adverbs were retained. Coherence and perplexity values were generated to help assess the performance of each model. The LDA outputs included generating the 10 most discriminative words for each topic; the weighting of each word within the allocated topic; and, for the *gensim* LDA model, a computer-based visualization (pyLDAvis [version 2.1.2]) that demonstrated the words for each topic and the degree of overlap between topics [66]. This visualization could also be used to show varying values of alpha and beta, the balance between words per topic and topics per document.

For the embedding-based method, no preprocessing of the text or prespecified number of topics was required because the Top2Vec algorithm calculates the number of topics contained within the corpus.

### Sentiment Analysis to Evaluate Effectiveness

Two widely used lexicon-based methods—TextBlob (version 0.15.3) [67] and Valence Aware Dictionary and Sentiment Reasoner (VADER; version 3.3.2) [68]—were compared and the strengths and limitations of both identified. The original cleaned *TextOnly* field was selected for the sentiment analysis because this contained only the responses to the posts. Word counts were calculated for each post. Capitalization, punctuation, and stop words were retained for this part of the analysis because each can contribute meaning or intensity to the analysis. TextBlob [67] calculates values for polarity and subjectivity for each post. The lexicon it uses derives from a separate library in the Natural Language Toolkit. It focuses on adjectives from customer product reviews that have been tagged by humans for polarity and subjectivity. Subjectivity analysis assesses how objective or subjective the text is, whereas polarity classification determines whether the text is positive or neutral. It uses the sentiment lexicon to assign scores for polarity and subjectivity for each word, which are then averaged out using a weighted average to provide an overall sentence sentiment score. Basic statistics were generated for both values, and the numerical polarity score was converted to categorical values of positive (>0), neutral (0), and negative (<0). Plots showing the distribution and the relationship between the polarity and subjectivity scores were generated.

The methods behind the design of the VADER library make it possibly a better choice for sentiment analysis of social media–type posts than TextBlob [69]. Rather than calculating the polarity and subjectivity of a post, it scores each post on 4 aspects: positive, negative, neutral, and compound. The positive, negative, and neutral scores represent the proportions of the post that fall in these categories. The compound score is calculated from the other 3 scores, normalized to a value between –1 and 1, and represents the overall sentiment of the post [68]. The lexicon VADER uses is based on general language rather than reviews [70] and contains approximately 7500 words.

Although the basic sentiment is calculated on the individual words, VADER looks at the whole text and can take negations into account [71]. This can help to give a balanced assessment when the post contains contradictory words out of context. This approach is intended to take into account some of the characteristics often seen in SGOPE data where features such as repeated punctuation or capital letters can be used to signify stronger sentiment [68].

The VADER lexicon is easily modified. After reviewing the positive and negative words it had identified from a sample of posts at each end of the sentiment spectrum, we modified the lexicon, removing the positive words *credit*, *free*, *accepted*, and *approval* because these words were frequently included in spam posts. We also added frequently mentioned effects to the negative lexicon, including *headache*, *jittery*, *rash*, *tired*, *harmful*, *disappointed*, *sleepy*, *nightmare*, and *intolerable*. In addition, we modified the positive lexicon to include *awake*, *focus*, *concentrate*, *normal*, *productive*, *helped*, *grateful*, *miracle* and *lifesaver*.

The results from each method were then compared against each other.

### Linguistic Analysis

We extracted the narrative fields from each post to form a corpus, which was then imported into Sketch Engine [72], a corpus linguistics tool. Each token was assigned a POS tag from the English TreeTagger POS tagset with Sketch Engine modifications [72]. Using the English Web corpus 2020 as a reference corpus [73], we generated lists of the top 1000 keywords, key terms, and n-grams specific to the dataset to help identify both themes and examples of causal text. N-grams are sequences of words, numbers, or symbols that appear in a specific order within the text and are helpful in identifying commonly used phrases of up to *n* words within the corpus [74]. For each word or term in the lists, we recorded its frequency in the focus corpus, the number of posts it appeared in, and a calculated score based on its relative frequency in each corpus. We then classified the top 100 highest-scoring keywords and key terms into themes and summarized the results to see how this technique compared to the topic modeling. N-grams that indicated a possible cause and effect or temporal dimension were identified. Combining these selected n-grams with concordance techniques revealed specific relevant sentences that expressed the poster’s understanding of these sequential events.

### Ethical Considerations

Ethics approval for the study was granted by the University of Warwick (BSREC Ref 11/19-20) in October 2019. No personally identifiable information other than the online “user handle” was included in the data collection, and this was removed and replaced with a unique ID for each post as part of the cleaning and preparation process.

## Results

### Descriptive

The cleaned dataset contained 68,559 records from a 6-year period (2011-2016). A total of 790 unique top-level sites were identified, with the number of posts per site ranging from 25,355 to 1. Reddit was the largest overall source, with 36.98% (25,355/68,559) of the posts from 213 subreddits, each of which represents a separate community. Of the 213 subreddits, 5 (2.3%) contributed >1000 posts, with the largest being the *afinil* subreddit (n=12,870, 18.77% posts). Post lengths ranged from 1 to 1577 (mean 100.4, SD 100.86; IQR 34-132) words. The *TextOnly* field comprised 7.99 million tokens, 6.84 million words, 104,565 unique words, and 388,516 sentences. Parsing the site or forum URLs revealed 166 separate health conditions. Multimedia Appendix 1 shows analysis by the number of posts posted to the top 10 condition-specific sites. This does not assume that the specified condition was the primary or sole condition of the poster but rather reflects the poster’s choice in selecting where to post their contribution.

### Topic Modeling

#### Overview

First, using the *gensim* LDA library, initial parameters were set to 8 topics (as per the earlier themes identified [21]) and 50 iterations. The default output is the top 10 words per topic, together with the weighting of each word within the topic. Although the returned topic word lists could all be seen to relate to the poster’s experience, they did not seem to be clearly distinguishable from each other. The visualization (Multimedia Appendix 2) indicates a substantial overlap of topics 1 to 4, which between them represented 72.7% (49,842/68,559) of the tokens.

**Figure 1:**
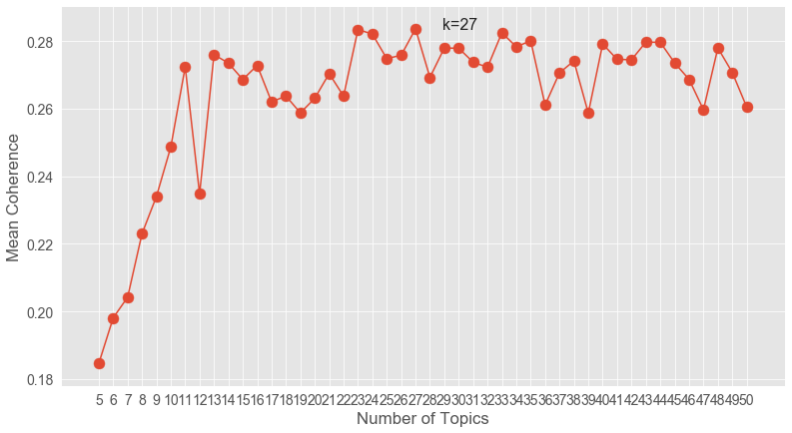
Coherence testing model (range 5-50).

Coherence model testing (Figure 1) using the NMF method (range 5-50) suggested that the optimal number of topics was 27; therefore, we ran the model again with varying numbers of iterations across the data.

#### Gensim LDA

Running the LDA model with parameters of 27 topics and 200 passes (Multimedia Appendix 3) showed a clearer distribution of topics, but there was still a substantial degree of overlap of topics 1 to 6. Increasing the number of passes to 1000 did not seem to significantly improve the visual evaluation (Multimedia Appendix 4), although it took >5 times as long to run.

**Figure 2:**
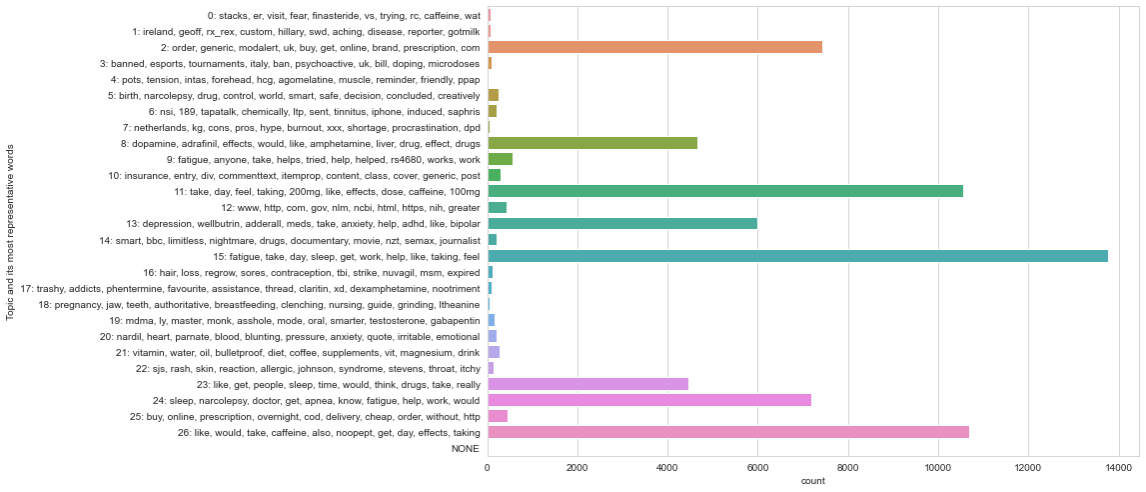
Latent Dirichlet allocation model: 27 topics.

**Figure 3:**
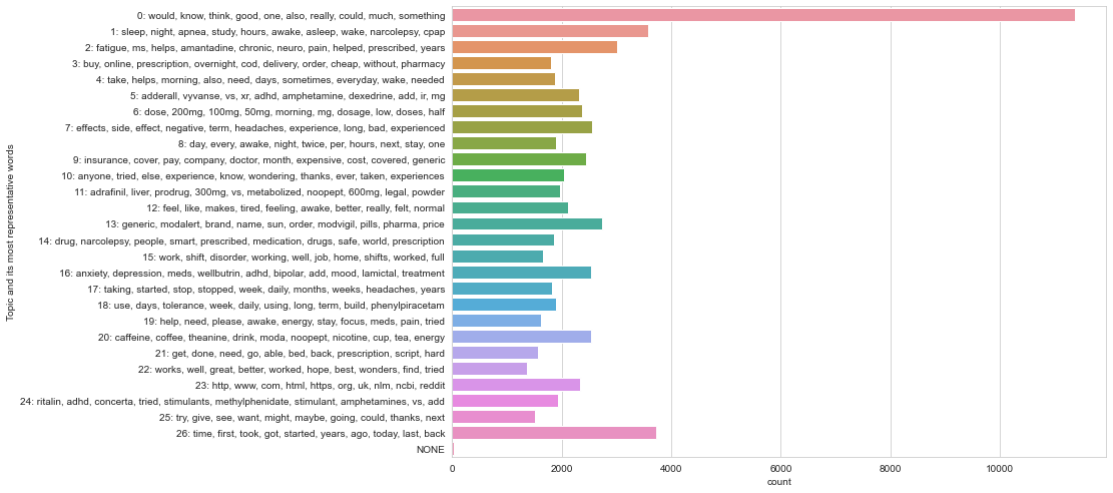
Nonnegative matrix factorization model: 27 topics.

Although both visualizations show some distinct topic circles that are not overlapped by others, the categorization of the topics into themes was not possible because most of them could have multiple interpretations. The top 10 topic words for each of the 27 topic models and the attempted mapping are shown in Figures 2 and 3.

In terms of the processing load, the timings of the *gensim* LDA models were impacted far more by the number of iterations through the data than the number of topics selected, with the simplest configuration—8 topics and 50 iterations—taking 32 minutes, 27 topics and 200 iterations taking 2 hours 16 minutes, and 27 topics and 1000 iterations taking 11 hours and 6 minutes. Adjusting the memory handling parameters reduced the processing time significantly (13 min, 1 h 44 min, and 8 h 13 min, respectively) but gave the highest coherence score to a model with just 2 topics and 10 passes, which did not seem a plausible result.

#### Sklearn LDA and NMF Methods

Running the same 27-topic model with the *sklearn* library enabled a direct comparison of the LDA and NMF methods. Multimedia Appendix 5 presents a comparison of the top 10 words per topic and the number of posts each model classified as belonging to each topic, together with the percentage of the corpus per topic in descending order for each method. It also includes our evaluation of the theme that the topic words most closely indicated. As with the earlier *gensim* LDA models, trying to map each of the returned topic word lists to the identified themes was complicated by the degree of overlap in most of the lists. The bar graphs (Figures 2 and 3) show that the NMF method returned topics that were distributed slightly more evenly throughout the corpus, whereas the LDA version identified some topics that were much less represented. The *sklearn* LDA model allocated 94.45% (64,753/68,559) of the posts to just 8 (30%) of the 27 topics; the remaining 19 (70%) topics each represented <1% (3806/68,559) of the posts. In comparison, the largest NMF topic was assigned to 16.6% (11,381/68,559) of the posts, with the remaining 26 ranging from 5.4% (3702/68,559) to 2% (1371/68,559) of the posts. Future work could look at going back to the posts included in some of the smaller topics to assess their relevance to the research question.

Mapping the topics found by both models, even at a superficial level, to distinct themes was problematic. For the *sklearn* LDA model, only 26% (7/27) of the topics could be mapped to the general themes. The NMF model was slightly more interpretable with 52% (14/27) of the topics that could be seen as relating to themes.

#### Top2Vec Library

The Top2Vec library demonstrated substantially faster performance compared to the LDA method. By default, it returns the number of detected topics, the top 50 words per topic, and the number of posts per topic. The optimal DeepLearn parameter took 2 hours 15 minutes to generate 367 topics from the dataset, while the Learn parameter took 19 minutes to generate 566 topics.

The results from the DeepLearn model were used for analysis. The percentage of posts per topic ranged from 2.94% (2017/68,559) in the largest group to 0.07% (45/68,559) in the smallest. Overall, 70% (257/367) of the posts could be mapped to either the P1 themes or the codes used during the thematic analysis. The P1 study refer to the previous part of the study where we compared a sample of 260 posts using a qualitative analysis with a basic NLP or corpus [21]. In total, 186 (50.7%) of the 367 topics representing 38,637 (56.36%) of the 68,559 posts could be mapped to the P1 themes. A further 71 (19.3%) of the 367 topics representing 15,557 (22.69%) of the 68,559 posts were mapped to the codes.

In total, 110 (30%) of the 367 topics representing 14,345 (20.92%) of the 68,559 posts were initially categorized as being uninterpretable without taking a deeper look at the specific posts. Of the 367 topics, 31 (8.4%; 3913/68,559, 5.7% posts) combined multiple themes and were classed as mixed; 50 (13.6%; 7019/68,559, 10.24% posts) were uninterpretable and were labeled unclear; and 29 (7.9%; 3413/68,559, 5% posts) contained words indicating that the topics related to possible spam posts.

### Sentiment Analysis

The TextBlob library returns values for both polarity and subjectivity. Of the 68,559 posts, the initial results for polarity were as follows: 47,282 (69%) positive, 6229 (9.09%) neutral, and 15,048 (21.95%) negative. The polarity scores extended across the whole range from −1 to +1 (mean +0.1003). The subjectivity scores also covered the entire range from 0 to +1 (mean +0.4638).

Using the previously mentioned parameters of positive (>0), neutral (0), and negative (<0), the initial results returned from the standard VADER analysis were 64.03% (43,898/68,559) positive, 6.7% (4592/68,559) neutral, and 29.27% (20,070/68,559) negative. Modifying the lexicon yielded the following results: 65.01% (44,610/68,559) positive, 6.44% (4417/68,559) neutral, and 28.49% (19,533/68,559) negative. The compound score values ranged −0.9991 to +0.9997 (mean +0.2825). The distribution is shown in Table 1.

**Table 1:** Basic statistics for the extended VADER analysis (n=68,559).

		Compound	Positive	Neutral	Negative
Scores, mean (SD; min-max)		0.28250790 (0.61562543; –0.99910000 to 0.99970000)	0.11785168 (0.09204523; 0.00000000-1.00000000)	0.81442440 (0.10185110; 0.00000000-1.00000000)	0.06772396 (0.06403353; 0.00000000-0.67000000)
**Percentile values**					
	25%	–0.1779	0.0590	0.7590	0.0120
	50%	0.4515	0.1070	0.8200	0.0580
	75%	0.8407	0.1600	0.8760	0.1010

**Figure 4:**
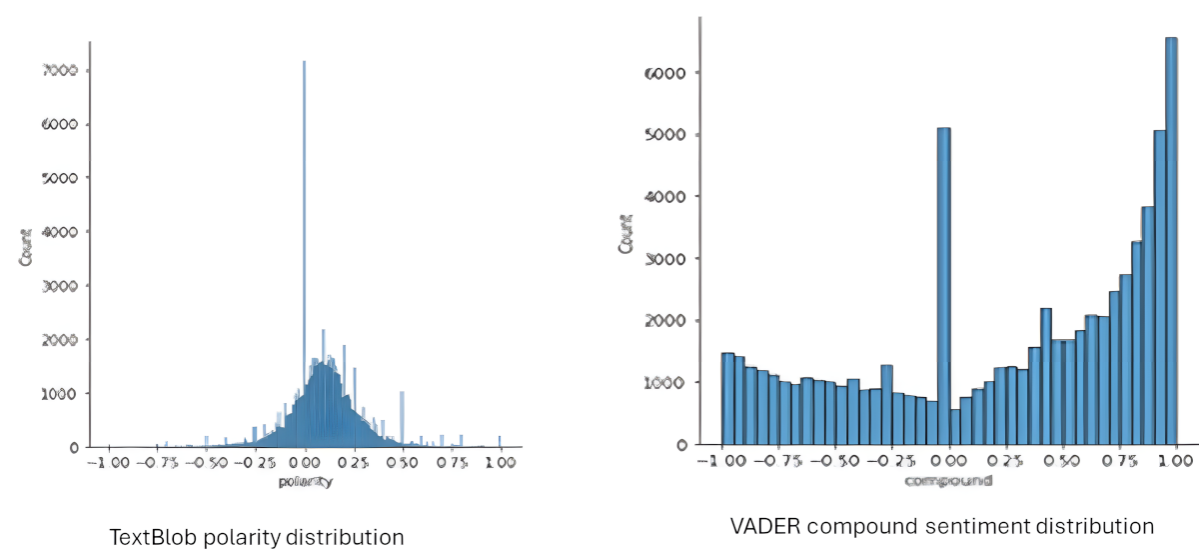
TextBlob and Valence Aware Dictionary and Sentiment Reasoner (VADER) distributions.

Although the results from both Vader and TextBlob methods were similar, with both showing a majority of posts being assessed as positive, comparing the distribution shape of the sentiment values between the methods showed distinct differences. Both are skewed toward the right, indicating the positive mean value; however, TextBlob showed a normal type of distribution of polarity apart from those posts classified as neutral, whereas Vader showed a similar peak at 0 but seemed to assess more of the posts as being at the extremes of the available range (Figure 4).

**Figure 5:**
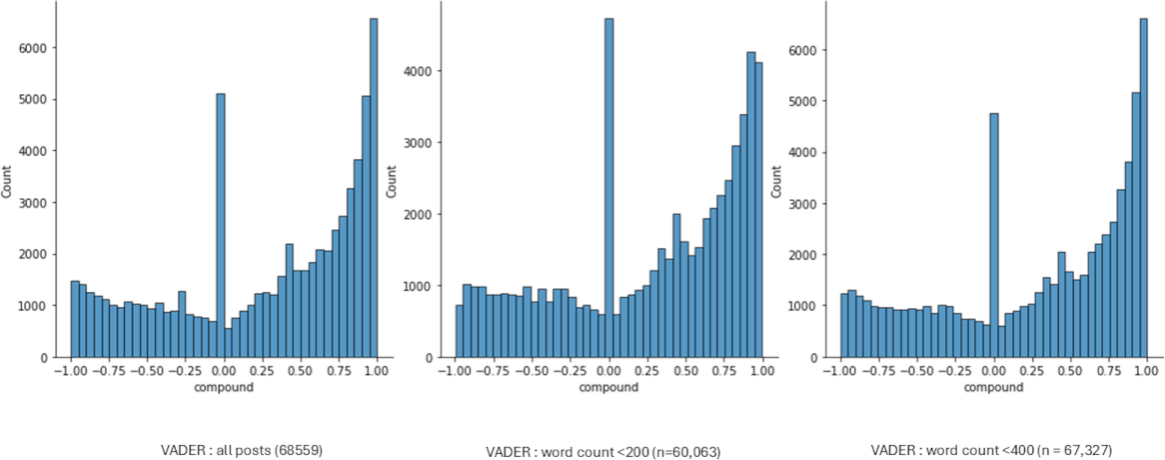
The impact of word count on sentiment. VADER: Valence Aware Dictionary and Sentiment Reasoner.

The average word count of the 10 highest-rated posts based on the VADER analysis was 704, and that of the lowest-rated posts was 1095. For TextBlob, the average word count of the 10 highest-rated posts was 39, and that of the lowest-rated posts was 23. VADER is reported as performing better on short texts [68]. The P3 dataset (total 68,559 posts) contained 1232 posts with a word count of >400 and 8496 posts longer than 200 words. However, running VADER again on the reduced datasets showed little difference in the percentages of posts rated in each category (Table 2; Figure 5).

**Table 2:** Valence Aware Dictionary and Sentiment Reasoner results from limiting post length.

	All posts, standard (n=68,559)	All posts, extended (n=68,559)	<400 words, extended (n=67,327)	<200 words, extended (n=60,063)
Compound scores, mean (SD; IQR)	0.2658 (0.613580; –0.2040 to +0.8250)	0.2819 (0.61587235; –0.1794 to +0.8404)	0.2816 (0.609968; –0.1779 to +0.8438)	0.2658 (0.587878; –0.1655 to +0.7984)
Positive, n (%)	43,898 (64.03)	44,586 (65.03)	43,781 (64.18)	38,546 (64.18)
Neutral, n (%)	4592 (6.70)	4416 (6.44)	4416 (6.56)	4414 (7.35)
Negative, n (%)	20,070 (29.27)	19,557 (28.53)	19,130 (28.41)	17,103 (28.48)

### Corpus Linguistics

Using the corpus linguistic tool Sketch Engine, we generated 1000 key n-grams specific to the SGOPE corpus, identifying many phrases that could suggest a form of causality. Attempting to map these key n-grams to the individual themes was problematic. Unlike the key words and terms, only 16 (16%) of the top 100 n-grams specific to the corpus could be directly mapped to themes. A full analysis would require looking at the n-grams in the context of the post. However, the key n-grams are helpful in detecting expressions of causality. Unlike the individual words, all of which have a POS tag that can indicate tense, n-grams are combinations of words. It was possible to label many of them as relating to past, present, or future tense or as indicating possible belief. Examples are shown in Table 3.

**Table 3:** Key n-grams indicating possible belief.

Key n-gram	Frequency (n=68,559), n	Total number of documents including the phrase (n=68.559), n	Score (relative frequency compared to the reference corpus)	Theme	Tense
keep me awake	406	396	50.0	Effect	Present
works for me	408	398	49.2	—^a^	Present
i have found	458	440	48.8	—	Past
but it does	488	485	48.3	—	Present
i find that	403	388	46.1	—	Present
was able to	610	579	46.0	—	Past
that i can	474	460	45.4	Outcome	Present
i felt like	396	377	45.2	Effect	Past
i find it	407	400	44.4	—	Present
gave me a	395	389	44.3	—	Past
in my experience	377	365	43.8	—	N/A^b^
because i have	381	380	42.6	—	Present
because i was	377	363	41.2	—	Past
because of the	576	561	34.7	—	N/A
and i think	368	363	32.3	—	Present
in my opinion	301	293	29.7	—	N/A
and it seems	258	257	29.7	—	Present
i have noticed	242	235	29.1	—	N/A
but i feel	241	237	28.7	—	Present
it gives me	230	226	27.9	—	Present
to kick in	225	217	27.9	Effect	N/A
seems to work	229	226	27.6	—	Present
it seems to be	237	237	27.4	—	Present
has helped me	225	219	27.2	—	Present
because i do	236	233	27.1	—	Present
effect on me	216	212	26.9	Effect	N/A
me feel like	220	216	26.9	Effect	N/A
it gave me	218	213	26.7	Effect	Past
changed my life	216	209	26.5	Outcome	Past
but it seems	231	231	26.3	—	Present
gives me a	216	210	26.3	Effect	Present
think it is	255	247	26.3	—	Present
as soon as i	227	223	25.9	—	Present
i can say	229	218	25.6	—	Present
it does help	205	204	25.6	—	Present
for me is	212	208	25.5	—	Present
i still feel	206	200	25.4	Effect	Present
my experience with	204	201	25.0	—	N/A
and i know	228	225	24.8	—	Present
thought i was	211	204	24.7	—	Past
thought it was	237	233	24.6	—	Past
and it helps	196	194	24.4	—	Present
know if i	208	204	24.3	—	N/A
i felt like i	198	188	24.1	Effect	Past
i found it	209	202	24.0	—	Past
i thought it	229	227	23.9	—	Past
seems to have	242	234	23.5	—	Present
it helps with	185	183	23.2	—	Present
it has helped	187	185	23.2	—	Past
it seems that	232	227	23.2	—	Present
i know this	200	197	23.2	—	Present
feel like it	190	186	22.9	—	Present
because of my	191	188	22.9	—	N/A
am able to	189	178	22.9	—	Present
great for me	182	182	22.8	—	N/A
i can sleep	181	177	22.8	Effect	Present
i started to	197	186	22.8	—	Past
and it worked	186	186	22.7	Effect	Past
have found that	198	195	22.7	—	Past
give you a	228	226	22.7	—	N/A
and i felt	188	184	22.6	Effect	Past
it wears off	176	172	22.2	Dosage	N/A
a huge difference	183	180	22.2	Effect	N/A
better for me	177	176	22.2	—	N/A
this is a	642	627	22.2	—	Present
i found out	187	181	21.7	—	Past

^a^Could not be mapped.

^b^N/A: not applicable.

The n-gram “have found that” was shown to be indicative of causal expression in the exploratory study [21]. Using it on the P3 dataset and filtering out any of the sentences that did not explicitly mention modafinil or one of its name variants in the concordance sentence returned the examples presented in Textbox 2.

**Concordance examples for the n-gram “have found that.**

**Tolerance**

• “I have been on Nuvigil for about 2 years now, and I have found that I have to skip my medication at least one day per week in order to not lose its effectiveness.” [Post ID 6289]

**Side effects**

• “I have found that I get visuals from modafinil anyways, for the first few hours of it’s effects I have mild visuals and a solid body load.” [Post ID 7711]

• “After taking modafinil 200mg next day i have found that i have a skin rash on the right hand and itchy skin on both hands.” [Post ID 26,660]

**Dosage**

• “Forgetting and False Memories I am on Nuvigil, and I have found that I become a ‘zombie’ when they have my dosage too high.” [Post ID 29,323]

**Other Intervention, but effect or outcome**

• “I have found that I have been able to reduce my Prozac dosage while taking Provigil.” [Post ID 53,387]

**Outcome**

• “I also have found that I am much more confident since started on provigil (200mg/day).” [Post ID 59,900]

**Comparison**

• “I have tried Adderall and Provigil and have found that I prefer a sister drug to the Provigil called Nuvigil, but my insurance company won’t pay for it so I’m stuck with the Provigil or Adderall.” [Post ID 67, 037]

The word sketch tool can be used to demonstrate the context of how any word or phrase is used within the corpus. Many of the key n-grams for this corpus relate to an observation the poster has made or an effect they have noticed in relation to the subject of their post. The most frequent key n-gram in the corpus is “in the morning,” which appears 3016 times in 2627 posts. Using the corpus query language to filter down to only those concordances that included modafinil in the same sentence returned 183 examples of dosage patterns, amounts, drug combinations, timing advice, and effects. As with the P1 study, posters reported how the standard dose can be excessive for some people [21]:

...my Dr prescribed starting dose of 200mg modafinil...once in the morning...with the instruction that if the200mg did not keep me awake that I should double the dose to 400mg once a day in the a.m...the 200mg was too much all at once...all it did was enhance the side effects to the point that I wasn’t able to notice if the medicine was doing what it was supposed to.because I was too busy cradling my cracked feeling skull and drinkn insane amounts of water. [Post ID 3209]

**Figure 6:**
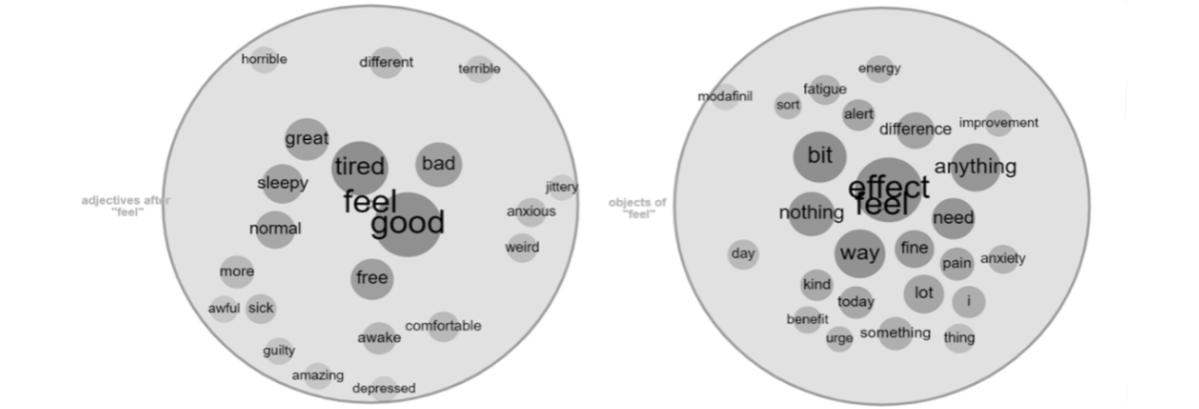
Most frequent adjectives and objects of “feel.”.

**Figure 7:**
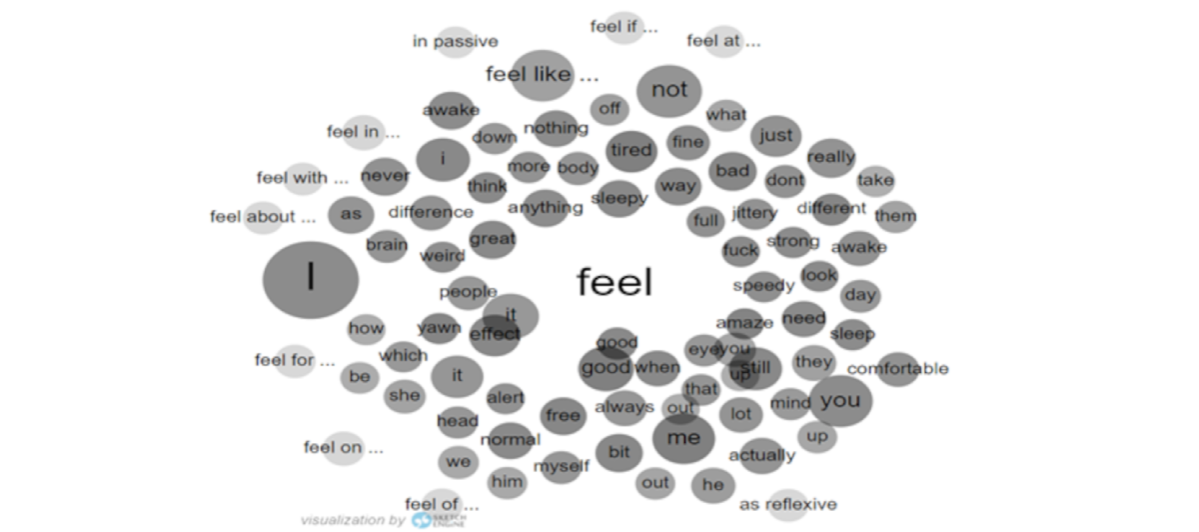
Word sketch of the verb “feel.”.

Another frequent lemma related to effectiveness in the n-grams is “feel,” which has been used by post writers in many ways. As a verb, it was used 22,767 times in the corpus. Splitting the occurrences into grammatical categories, as shown in Table 4, highlights the categories, some of the most frequent examples of each phrase from the corpus, and the number of occurrences for each category. A visual representation of the most frequent adjectives and objects associated with the verb “feel” is shown in Figure 6, while Figure 7 displays the most frequent collocates. The size of each circle represents the frequency of the collocate. Of note, “good” is the most prominent adjective collocate of “feel,” supporting the hypothesis that modafinil is perceived as effective by many of the posters. The full list of collocates of “feel,” together with their frequencies in the corpus, is available in Multimedia Appendix 6.

Feeling normal was identified as being an important outcome for some posters in the earlier study [21]. Textbox 3 presents examples of n-gram concordances for “makes me feel,” filtered by “normal.”

**Table:** 

Grammatical categories	Examples	Frequency (n=68,559), n (%)
pronominal subjects of “feel”	I feel, you feel, made me feel, it feels	12,026 (17.54)
modifiers of “feel”	Don’t feel, I still feel, I just feel, really feel	6842 (9.98)
adjectives after “feel”	feel better, feel tired, feel worse, feel great, feel sleepy, feel normal	5342 (7.92)
objects of “feel”	feel the effects, feel a bit, felt nothing	4354 (6.61)
prepositional phrases associated with “feel”	feel like, feel in, feel on, feel though	2163 (3.15)
subjects of “feel”	I feel, my body feels, I don’t feel	2032 (2.96)
pronominal objects of “feel”	feel it, you feel you, feel myself	689 (1)
complements of “feel”	feel a lot better, felt it more, felt a bit weird	289 (0.42)
“wh-” words following “feel”	feel when, feel what, I feel that, feel how, feel normal which	179 (0.26)
“feel” and or	sleep and feel, yawning and feeling	150 (0.22)
“-ing” objects of “feel”	felt taking, felt amazing	81 (0.12)
particles after “feel”	feel up to it, feeling down,	74 (0.11)
infinitive objects of “feel”	it feels to be	37 (0.05)
particles after “feel” with object	feel hyped up, to feel out	19 (0.03)

**Concordance of “makes me feel” with “normal.”**

**Examples of n-gram concordances for “makes me feel,” filtered by “normal”**

- I have never noticed excessive energy or anything out of the ordinary; it just makes/make me/me feel/feel like a normal person would.
- Taking the whole thing almost makes/make me/me feel/feel normal for a while.
- Anything that makes me less sleepy makes/make me/me feel/feel more “normal” (i.e., less tired), and not high (course I am not shooting it in my arm or anything).
- While Modafinil *feels* like a some sort of drug-induced happiness, Zoloft actually makes/make me/me feel/feel naturally normal and happy.
- Cheers. :) I am on Modafinil which makes/make me/me feel/feel normal most of the time. @Nicole – I’m showing my age, but as a student it was ProPlus every time for me!
- It just makes/make me/me feel/feel closer to normal.
- At first I did feel speedy but now it just makes/make me/me feel/feel normal (ish)!!
- Doesn’t jack me up or give me jitters - just makes/make me/me feel/feel as “normal people normal” as I can imagine.
- My epileptologist has just put me on nuvigil for sleepiness and it really helps, there is only a day here and there it doesn’t but it’s awesome now most of the time I have the energy that my family has (2 kids) doesn’t make me hyper just honestly makes/make me/me feel/feel more normal.
- I take Nuvigil, and, unlike stimulants, it just makes/make me/me feel/feel normal without the waves of crippling exhaustion or a crash at the end of the day.
- Nuvigil makes/make me/me feel/feel like a normal person again and without it, my quality of life is severely decreased.
- I love nuvigil and it makes/make me/me feel/feel “normal” and have a “normal” life but somedays I feel like I could use another pill and if its *safe* to take it twice a day then that may help me ALOT!!
- I have read posts where people talk about feeling revved up from it but for me it just makes/make me/me feel/feel normal.
- The provigil makes/make me/me feel/feel normal.
- It just makes/make me/me feel/feel normal which is perfect...no jitters.
- It makes/make me/me feel/feel normal.
- It makes/make me/me feel/feel pretty normal like I used too.
- I usually take it around noon at work during the week and it makes/make me/me feel/feel normal, and I can get through the rest of the day.
- It makes/make me/me feel/feel normal.
- I am taking 200mg an hour before work and it makes/make me/me feel/feel normal.I try not to take it every day, but it definitely helps...makes/make me/me feel/feel normal almost.
- I’ve now been feeling like it makes/make me/me feel/feel more “normal” (normal energy & focus) for a few hours past my dose (8am and 2pm) and the other times are like a complete drop in energy, not even normal tired....just SO exhausted.
- (It wasn’t my first choice.) The only thing that makes/make me/me feel/feel close to normal is use of stimulants such as Nuvigil, but those give me serious insomnia.
- I hate that a pill/pills makes/make me/me feel/feel normal.
- It doesn’ make me feel buzzed or jittery, it just makes/make me/me feel/feel “normal.”

### Comparison With Existing Trial Evidence

The effectiveness of modafinil suggested by this study contrasts with the existing RCT and systematic review evidence that is used to determine treatment pathway options for clinicians [28]. Rather than searching for every review or RCT of modafinil, we used Cochrane reviews as a comparison. Cochrane reviews critically appraise individual trials, are recognized as providing high-quality assessment and evidence synthesis, and are also used to contribute to the development of clinical guidelines [75]. As of May 2021, a search of the Cochrane Library [76] showed that there were 16 published Cochrane reviews for various conditions that included the term modafinil in the title, abstract, or keywords. To compare the findings, we extracted the authors’ evidence summaries, the quality assessments of the evidence, and suggestions for addressing the remaining uncertainties relevant to this project (Multimedia Appendix 7). All reviews were inconclusive, with either insufficient [77-85] or low-quality [86-92] evidence of effectiveness. One of the main findings of this study was that although modafinil is only currently licensed by the National Institute for Health and Care Excellence for a single condition within the United Kingdom, posters were finding it effective for a wide range of conditions, including central disorders of hypersomnolence, multiple sclerosis, attention-deficit disorder and attention-deficit/hyperactivity disorder, social anxiety, depression, sleep-related breathing disorders, general fatigue, myalgic encephalomyelitis and chronic fatigue syndrome, and fibromyalgia (Figure 1). Other conditions for which modafinil was used included cancer fatigue, traumatic brain injury, diabetes, epilepsy, autoimmune conditions, pain, irritable bowel syndrome, hepatitis C, and poststroke fatigue. Multimorbidity was a regular feature.

## Discussion

### Principal Findings

Although a range of positive and negative experiences were reported, our analysis indicates that posters found modafinil effective for their symptoms with similar levels of effectiveness found across all methods. Similar themes were identified by both qualitative and computational analyses. Difficulties in obtaining a prescription or acquiring modafinil were common. All topic-modeling methods returned topics containing words that clearly related to and could be mapped to the themes and subcodes from the earlier qualitative study [21]. Linguistic analysis identified expressions of causal belief.

The overall methodology of the study was designed so that it can be applied to other health-related research questions that use unstructured data. The principles underlying the methods used in this study have shown that they can be used inductively on large volumes of unstructured text to extract the themes, sentiment, and expressions of perceived causality.

As an inductive and iterative method, topic modeling shows potential for scaling up qualitative analysis [43,61,93] when working with large volumes of data. The requirement of both the LDA and NMF methods for a defined number of topics to be determined before running the models is problematic. Previous comparisons of findings from both manual coders and NMF topic modeling found that neither group could agree on the ideal number of topics [61]. Using the Top2Vec method had the advantage that it did not require a predetermined number of topics or themes to be specified. The Top2Vec embedding-based method was more effective in eliciting topics that mapped to those previously identified through qualitative analysis [21]. A possible disadvantage of this model is that, depending on the dataset, it may return too many topics [94], but this can be mitigated in a later version of the model through the use of hierarchical topic reduction [64].

Previous studies have commented on how lexicon-based tools trained on general language do not perform as well on health-related text [3]. Although lexicon-based sentiment analysis can provide an accurate assessment of text that contains words that express a strong positive or negative sentiment, posts that do not contain many of these predefined words are harder to evaluate. One of the features of the informal nature of SGOPE data is that the writers assume that readers can readily infer the affective reaction they are describing. Descriptive phrases such as “I could go back to work” or “It gave me a headache” suggest the effect of the event but would be viewed as neutral statements by most sentiment analysis models. Developing lexicons that are more relevant to health outcomes would improve and refine the results.

The inclusion of linguistic analysis added a depth of understanding to the findings that would not have been possible with a pure NLP approach [38,39]. The reported rapid onset of the effect of modafinil, whether positive or negative, together with the temporal sequencing, allowed the identification of text indicating perceived causality.

Unsupervised methods align more with the inductive approach of qualitative studies and are shown to be effective for exploring SGOPE data. Although topic modeling has not yet been widely used within health research, previous studies have shown how it can be used to generate findings in a similar fashion to grounded theory [21,42]. Both topic modeling and the extraction of keywords, key terms, and key n-grams identify what is being spoken about but not how the word or phrase is used in context. Combining NLP with corpus linguistics draws on the strengths of both disciplines [38,39] and allows the researcher to identify the content that is most relevant to the research question [95].

This research could be extended in a variety of ways that could be used to improve health outcomes. Extending the case study approach, these could include extracting features such as dosage detail and treatment duration, examining more granular topics, further refining the lexicons used for sentiment analysis, and conducting tense analysis of POS tags of modafinil or other interventions. Combining NLP with linguistics on large quantities of unstructured data could be a valuable source both to identify “off-label” indications and obtain a deeper understanding of the outcomes that patients and their families prioritize and how they are managing their conditions. In terms of methodological development, these methods could also be applied to many different types of unstructured text sources, such as qualitative interview transcripts or the free-text sections of clinical notes.

### Strengths and Limitations

The use of unsupervised methods allows for an inductive approach to analysis, and the comparison of findings from multiple methods with those from the exploratory dataset is a strength of this study. SGOPE data analysis relies on the poster’s self-description of their condition, which may include self-diagnosis rather than a clinician’s assessment. The reporting of symptoms and outcomes may not be as accurate or complete as it could be, although this limitation could apply to any form of self-reported data, whether collected in a trial, clinical encounter, or on the web. Self-reported data, particularly regarding hard-to-measure factors such as fatigue and cognition, are subjective but generally reflect the normative value of the patient. The natural, nonclinical language used in informal texts may contain valuable, unexplored, or overlooked information relevant to clinical or research purposes [96], but it can also contain spelling or grammatical errors and inappropriate slang or colloquialisms that pose challenges for NLP methods [97]. Keyword comparison with a reference corpus was found to be effective in identifying such terms and common misspellings.

SGOPE data have several known strengths and limitations [98-100] as a single data source. Using multiple data sites enhanced the representativeness and validity of the sample and reduced the potential for demographic bias and emotional contagion (18), while mitigating the impact of spam or nongenuine posts through the cleaning process. We do recognize the limitation of only including posts written in English. Although social media use is widespread, those who create posts represent a self-selected subset of users, with only 10% estimated to be active posters, while 90% read other users’ posts without contributing their own comments [101].

### Conclusions

The study demonstrated the value of combining NLP and linguistic techniques for analyzing large quantities of unstructured text that can then be used as evidence of improved patient outcomes. In contrast to the current systematic review–based evidence, posters with a wide range of conditions found modafinil effective. The methods we used successfully identified the entities and topics contained in posts. The perceived experiences of causality and effectiveness were identified using 2 different methods. Our study indicates that this NLP- and linguistics-based approach can be used to look beyond the literal meaning of the words in posts, gaining an understanding of how posters assess the effectiveness of a health care intervention and the outcomes they value.

## Appendix

Multimedia Appendix 1 Postfrequency across condition-specific sites derived from parsing of data source URLs.

Multimedia Appendix 2 Latent Dirichlet allocation: 8 topics and 50 iterations.

Multimedia Appendix 3 Latent Dirichlet allocation: 27 topics and 200 iterations.

Multimedia Appendix 4 Latent Dirichlet allocation: 27 topics and 1000 iterations.

Multimedia Appendix 5 Comparison of the top ten words of the sklearn latent Dirichlet allocation and nonnegative matrix factorization topics (n=27).

Multimedia Appendix 6 Collocates and frequency of “feel”.

Multimedia Appendix 7 Cochrane library reviews including modafinil: May 2021.

## Abbreviations

LDA: latent Dirichlet allocation
NLP: natural language processing
NMF: nonnegative matrix factorization
POS: part-of-speech
RCT: randomized controlled trial
RWD: real-world data
SGOPE: spontaneously generated online patient experience
VADER: Valence Aware Dictionary and Sentiment Reasoner

## Data Availability

The code generated and analyzed during this study is available from the corresponding author on reasonable request.

## References

[1] Gohil S, Vuik S, Darzi A (2018). Sentiment analysis of health care tweets: review of the methods used. JMIR Public Health Surveill.

[2] Edo-Osagie O, De La Iglesia B, Lake I, Edeghere O (2020). A scoping review of the use of Twitter for public health research. Comput Biol Med.

[3] Zunic A, Corcoran P, Spasic I (2020). Sentiment analysis in health and well-being: systematic review. JMIR Med Inform.

[4] Walsh J, Dwumfour C, Cave J, Griffiths F (2022). Spontaneously generated online patient experience data - how and why is it being used in health research: an umbrella scoping review. BMC Med Res Methodol.

[5] Wong A, Plasek JM, Montecalvo SP, Zhou L (2018). Natural language processing and its implications for the future of medication safety: a narrative review of recent advances and challenges. Pharmacotherapy.

[6] Lafferty NT, Manca A (2015). Perspectives on social media in and as research: a synthetic review. Int Rev Psychiatry.

[7] Foufi V, Timakum T, Gaudet-Blavignac C, Lovis C, Song M (2019). Mining of textual health information from Reddit: analysis of chronic diseases with extracted entities and their relations. J Med Internet Res.

[8] Suresh S (2020). Patient-generated health data can provide value in clinical care, research settings. American Academy of Pediatrics News.

[9] Vilar S, Friedman C, Hripcsak G (2018). Detection of drug-drug interactions through data mining studies using clinical sources, scientific literature and social media. Brief Bioinform.

[10] Su C, Xu Z, Pathak J, Wang F (2020). Deep learning in mental health outcome research: a scoping review. Transl Psychiatry.

[11] Abbe A, Grouin C, Zweigenbaum P, Falissard B (2016). Text mining applications in psychiatry: a systematic literature review. Int J Methods Psychiatr Res.

[12] Demner-Fushman D, Elhadad N (2016). Aspiring to unintended consequences of natural language processing: a review of recent developments in clinical and consumer-generated text processing. Yearb Med Inform.

[13] Döbrössy B, Girasek E, Susánszky A, Koncz Z, Győrffy Z, Bognár VK (2020). "Clicks, likes, shares and comments" a systematic review of breast cancer screening discourse in social media. PLoS One.

[14] Kim SJ, Marsch LA, Hancock JT, Das AK (2017). Scaling up research on drug abuse and addiction through social media big data. J Med Internet Res.

[15] Number of social media users worldwide from 2017 to 2028. Statista.

[16] Social media fact sheet. Pew Research Center.

[17] Bour C, Ahne A, Schmitz S, Perchoux C, Dessenne C, Fagherazzi G (2021). The use of social media for health research purposes: scoping review. J Med Internet Res.

[18] Cesare N, Grant C, Nsoesie EO (2019). Understanding demographic bias and representation in social media health data. Proceedings of the Companion Publication of the 10th ACM Conference on Web Science.

[19] Golder S, Norman G, Loke YK (2015). Systematic review on the prevalence, frequency and comparative value of adverse events data in social media. Br J Clin Pharmacol.

[20] Frost J, Okun S, Vaughan T, Heywood J, Wicks P (2011). Patient-reported outcomes as a source of evidence in off-label prescribing: analysis of data from PatientsLikeMe. J Med Internet Res.

[21] Walsh J, Cave J, Griffiths F (2021). Spontaneously generated online patient experience of modafinil: a qualitative and NLP analysis. Front Digit Health.

[22] Greenhalgh T (2016). Cultural Contexts of Health: The Use of Narrative Research in the Health Sector.

[23] Drewniak D, Glässel A, Hodel M, Biller-Andorno N (2020). Risks and benefits of web-based patient narratives: systematic review. J Med Internet Res.

[24] McKenna B, Myers MD, Newman M (2017). Social media in qualitative research: challenges and recommendations. Inf Organ.

[25] Sackett DL, Rosenberg WM, Gray JA, Haynes RB, Richardson WS (1996). Evidence based medicine: what it is and what it isn't. BMJ.

[26] Kones R, Rumana U, Merino J (2014). Exclusion of 'nonRCT evidence' in guidelines for chronic diseases - is it always appropriate? The Look AHEAD study. Curr Med Res Opin.

[27] Ogilvie D, Adams J, Bauman A, Gregg EW, Panter J, Siegel KR, Wareham NJ, White M (2020). Using natural experimental studies to guide public health action: turning the evidence-based medicine paradigm on its head. J Epidemiol Community Health.

[28] Schlegl E, Ducournau P, Ruof J (2017). Different weights of the evidence-based medicine triad in regulatory, health technology assessment, and clinical decision making. Pharmaceut Med.

[29] EBM+: integrating diverse evidence in evidence-based medicine. University of Kent.

[30] Greenhalgh T (2020). Will COVID-19 be evidence-based medicine's nemesis?. PLoS Med.

[31] Anjum RL, Copeland S, Rocca E (2020). Conclusion: causehealth recommendations for making causal evidence clinically relevant and informed. Rethinking Causality, Complexity and Evidence for the Unique Patient.

[32] (2024). Real-world evidence. U.S. Food and Drug Administration.

[33] Schilsky RL (2017). Finding the evidence in real-world evidence: moving from data to information to knowledge. J Am Coll Surg.

[34] Miani C, Robin E, Horvath V, Manville C, Cave J, Chataway J (2014). Health and healthcare: assessing the real world data policy landscape in Europe. Rand Health Q.

[35] Averitt AJ, Weng C, Ryan P, Perotte A (2020). Translating evidence into practice: eligibility criteria fail to eliminate clinically significant differences between real-world and study populations. NPJ Digit Med.

[36] Bender E, Gebru T, McMillan-Major A, Shmitchell S (2021). On the dangers of stochastic parrots: can language models be too big?. Proceedings of the 2021 ACM Conference on Fairness, Accountability, and Transparency.

[37] Strubell E, Ganesh A, McCallum A Energy and policy considerations for deep learning in NLP. arXiv.

[38] Lee J (2019). Writing linguistic rules for natural language processing. Medium.

[39] Bender EM, Koller A (2020). Climbing towards NLU: on meaning, form, and understanding in the age of data. Proceedings of the 58th Annual Meeting of the Association for Computational Linguistics.

[40] Abdellaoui R, Foulquié P, Texier N, Faviez C, Burgun A, Schück S (2018). Detection of cases of noncompliance to drug treatment in patient forum posts: topic model approach. J Med Internet Res.

[41] Maier D, Waldherr A, Miltner P, Wiedemann G, Niekler A, Keinert A, Pfetsch B, Heyer G, Reber U, Häussler T, Schmid-Petri H, Adam S (2018). Applying LDA topic modeling in communication research: toward a valid and reliable methodology. Commun Methods Meas.

[42] Baumer EP, Mimno D, Guha S, Quan E, Gay GK (2017). Comparing grounded theory and topic modeling: extreme divergence or unlikely convergence?. J Assoc Inf Sci Technol.

[43] Spasic I, Button K (2020). Patient triage by topic modeling of referral letters: feasibility study. JMIR Med Inform.

[44] Greaves F, Ramirez-Cano D, Millett C, Darzi A, Donaldson L (2013). Use of sentiment analysis for capturing patient experience from free-text comments posted online. J Med Internet Res.

[45] Kerry R, Anjum RL, Copeland S, Rocca E (2020). Causal dispositionalism and evidence based healthcare. Rethinking Causality, Complexity and Evidence for the Unique Patient.

[46] Deaton A, Cartwright N (2018). Understanding and misunderstanding randomized controlled trials. Soc Sci Med.

[47] Anjum RL, Anjum RL, Copeland S, Rocca E (2020). Dispositions and the unique patient. Rethinking Causality, Complexity and Evidence for the Unique Patient.

[48] Edwards R Living with complexity and big data. Uppsala Monitoring Centre.

[49] Neeleman A, van de Koot H (2012). The linguistic expression of causation. The Theta System: Argument Structure at the Interface.

[50] Williamson J (2019). Establishing causal claims in medicine. Int Studies Philos Sci.

[51] Greenhalgh T, Snow R, Ryan S, Rees S, Salisbury H (2015). Six 'biases' against patients and carers in evidence-based medicine. BMC Med.

[52] Davies N (2015). Social media: the voice of the patient. Reuters Events.

[53] van Rossum G (1995). Python reference manual. Centrum Wiskunde & Informatica.

[54] jupyterlab. GitHub.

[55] Albalawi R, Yeap TH, Benyoucef M (2020). Using topic modeling methods for short-text data: a comparative analysis. Front Artif Intell.

[56] Khanbhai M, Anyadi P, Symons J, Flott K, Darzi A, Mayer E (2021). Applying natural language processing and machine learning techniques to patient experience feedback: a systematic review. BMJ Health Care Inform.

[57] Chen Y, Zhang H, Liu R, Ye Z, Lin J (2019). Experimental explorations on short text topic mining between LDA and NMF based schemes. Knowl Based Syst.

[58] Jang H, Rempel E, Roth D, Carenini G, Janjua NZ (2021). Tracking COVID-19 discourse on Twitter in North America: infodemiology study using topic modeling and aspect-based sentiment analysis. J Med Internet Res.

[59] Suri P, Roy NR (2017). Comparison between LDA and NMF for event-detection from large text stream data. Proceedings of the 3rd International Conference on Computational Intelligence & Communication Technology (CICT).

[60] Birks D, Coleman A, Jackson D (2020). Unsupervised identification of crime problems from police free-text data. Crime Sci.

[61] Bakharia A (2019). On the equivalence of inductive content analysis and topic modeling. Proceedings of the First International Conference on Advances in Quantitative Ethnography.

[62] Rehurek R (2010). gensim: python framework for vector space modelling. Machine Learning Open Source Software.

[63] Pedregosa F, Varoquaux G, Gramfort A, Michel V, Thirion B, Grisel O, Blondel M, Prettenhofer P, Weiss R, Dubourg V, Vanderplas J, Passos A, Cournapeau D (2011). Scikit-learn: machine learning in python. J Mach Learn Res.

[64] Angelov D Top2Vec: distributed representations of topics. arXiv.

[65] A guide on word embeddings in NLP. Turing.

[66] Sievert C, Shirley KE (2014). LDAvis: a method for visualizing and interpreting topics. Proceedings of the Workshop on Interactive Language Learning, Visualization, and Interfaces.

[67] Loria S TextBlob: simplified text processing. TextBlob.

[68] Hutto C, Gilbert E (2014). VADER: a parsimonious rule-based model for sentiment analysis of social media text. Proc Int AAAI Conf Web Soc Media.

[69] Bonta V, Kumaresh N, Janardhan N (2019). A comprehensive study on lexicon based approaches for sentiment analysis. Asian J Comput Sci Technol.

[70] Soma J Comparing sentiment analysis tools. Data Science for Journalism.

[71] Caren N (2019). Word lists and sentiment analysis. Neal Caren.

[72] Sketch Engine.

[73] enTenTen: corpus of the English web. Sketch Engine.

[74] What is an N-Gram?. MathWorks.

[75] Alper BS, Fedorowicz Z, van Zuuren EJ (2015). Limitations in conduct and reporting of cochrane reviews rarely inhibit the determination of the validity of evidence for clinical decision-making. J Evid Based Med.

[76] Cochrane library homepage. Cochrane Library.

[77] Ruthirakuhan MT, Herrmann N, Abraham EH, Chan S, Lanctôt KL (2018). Pharmacological interventions for apathy in Alzheimer's disease. Cochrane Database Syst Rev.

[78] Castells X, Cunill R, Pérez-Mañá C, Vidal X, Capellà D (2016). Psychostimulant drugs for cocaine dependence. Cochrane Database Syst Rev.

[79] Day J, Yust-Katz S, Cachia D, Wefel J, Tremont Lukats IW, Bulbeck H, Rooney AG (2022). Interventions for the management of fatigue in adults with a primary brain tumour. Cochrane Database Syst Rev.

[80] Dougall D, Poole N, Agrawal N (2015). Pharmacotherapy for chronic cognitive impairment in traumatic brain injury. Cochrane Database Syst Rev.

[81] Elbers RG, Verhoef J, van Wegen EE, Berendse HW, Kwakkel G (2015). Interventions for fatigue in Parkinson's disease. Cochrane Database Syst Rev.

[82] Mücke M, Cuhls H, Peuckmann-Post V, Minton O, Stone P, Radbruch L, Mochamat (2015). Pharmacological treatments for fatigue associated with palliative care. Cochrane Database Syst Rev.

[83] Koopman FS, Beelen A, Gilhus NE, de Visser M, Nollet F (2015). Treatment for postpolio syndrome. Cochrane Database Syst Rev.

[84] Day J, Zienius K, Gehring K, Grosshans D, Taphoorn M, Grant R, Li J, Brown PD (2014). Interventions for preventing and ameliorating cognitive deficits in adults treated with cranial irradiation. Cochrane Database Syst Rev.

[85] Pérez-Mañá C, Castells X, Torrens M, Capellà D, Farre M (2013). Efficacy of psychostimulant drugs for amphetamine abuse or dependence. Cochrane Database Syst Rev.

[86] Ortiz-Orendain J, Covarrubias-Castillo SA, Vazquez-Alvarez AO, Castiello-de Obeso S, Arias Quiñones GE, Seegers M, Colunga-Lozano LE (2019). Modafinil for people with schizophrenia or related disorders. Cochrane Database Syst Rev.

[87] Castells X, Blanco-Silvente L, Cunill R (2018). Amphetamines for attention deficit hyperactivity disorder (ADHD) in adults. Cochrane Database Syst Rev.

[88] Gibbons C, Pagnini F, Friede T, Young CA (2018). Treatment of fatigue in amyotrophic lateral sclerosis/motor neuron disease. Cochrane Database Syst Rev.

[89] Liira J, Verbeek JH, Costa G, Driscoll TR, Sallinen M, Isotalo LK, Ruotsalainen JH (2014). Pharmacological interventions for sleepiness and sleep disturbances caused by shift work. Cochrane Database Syst Rev.

[90] Ker K, Edwards PJ, Felix LM, Blackhall K, Roberts I (2010). Caffeine for the prevention of injuries and errors in shift workers. Cochrane Database Syst Rev.

[91] Candy M, Jones L, Williams R, Tookman A, King M (2008). Psychostimulants for depression. Cochrane Database Syst Rev.

[92] Annane D, Moore DH, Barnes PR, Miller RG (2006). Psychostimulants for hypersomnia (excessive daytime sleepiness) in myotonic dystrophy. Cochrane Database Syst Rev.

[93] Gonzalez G, Vaculik K, Khalil C, Zektser Y, Arnold C, Almario CV, Spiegel B, Anger J (2022). Using large-scale social media analytics to understand patient perspectives about urinary tract infections: thematic analysis. J Med Internet Res.

[94] Egger R, Yu J (2022). A topic modeling comparison between LDA, NMF, Top2Vec, and BERTopic to demystify Twitter posts. Front Sociol.

[95] Isoaho K, Gritsenko D, Mäkelä E (2019). Topic modeling and text analysis for qualitative policy research. Policy Stud J.

[96] Rastegar-Mojarad M, Ye Z, Wall D, Murali N, Lin S (2015). Collecting and analyzing patient experiences of health care from social media. JMIR Res Protoc.

[97] Dirkson A, Verberne S, Kraaij W (2019). Lexical normalization of user-generated medical text. Proceedings of the Fourth Social Media Mining for Health Applications (#SMM4H) Workshop & Shared Task.

[98] Dalmer NK (2017). Questioning reliability assessments of health information on social media. J Med Libr Assoc.

[99] Wang Y, McKee M, Torbica A, Stuckler D (2019). Systematic literature review on the spread of health-related misinformation on social media. Soc Sci Med.

[100] Staccini P, Fernandez-Luque L (2017). Secondary use of recorded or self-expressed personal data: consumer health informatics and education in the era of social media and health apps. Yearb Med Inform.

[101] van Mierlo T (2014). The 1% rule in four digital health social networks: an observational study. J Med Internet Res.

