# Supplementary figures and images for: Combining Topic Modeling, Sentiment Analysis, and Corpus Linguistics to Analyze Unstructured Web-Based Patient Experience Data: Case Study of Modafinil Experiences
Source: J Med Internet Res. 2024 Dec 11;26:e54321. doi: 10.2196/54321 (PMC11669883; doi:10.2196/54321)

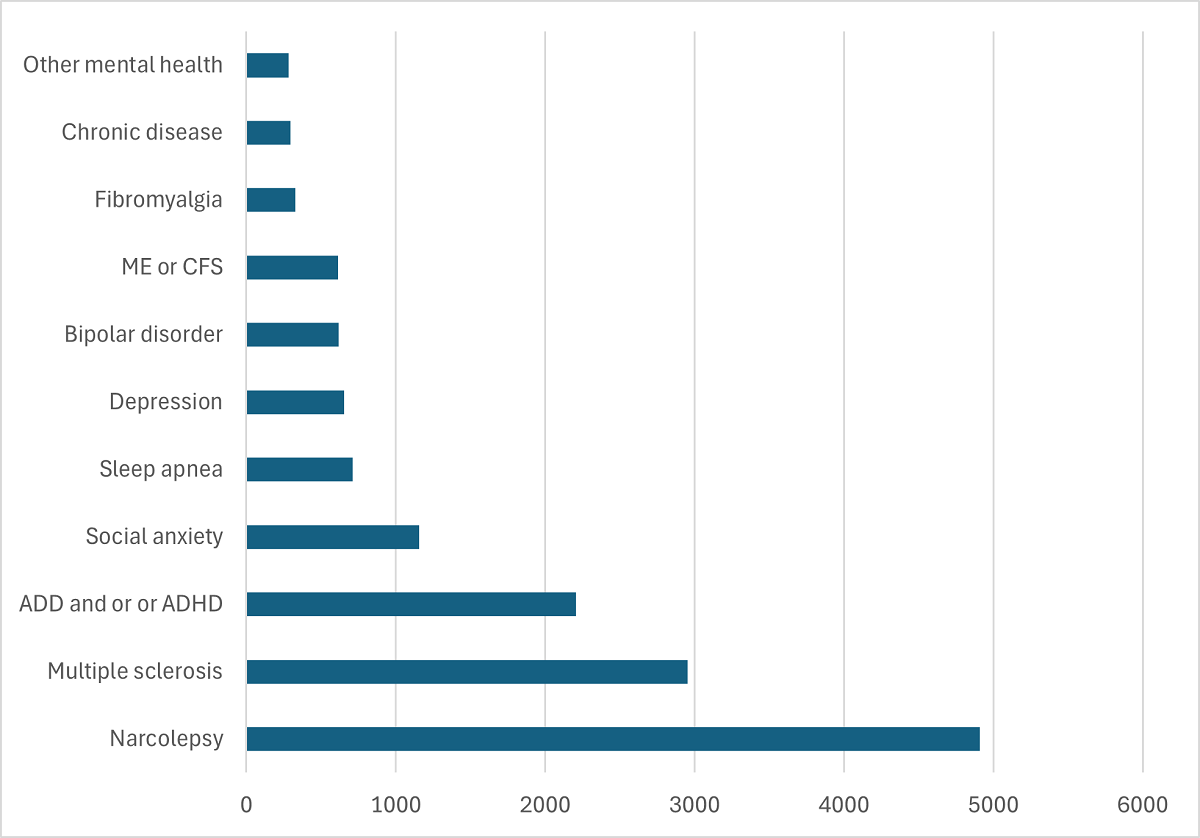

Supplement: Multimedia Appendix 1 [file jmir_v26i1e54321_app1.png]

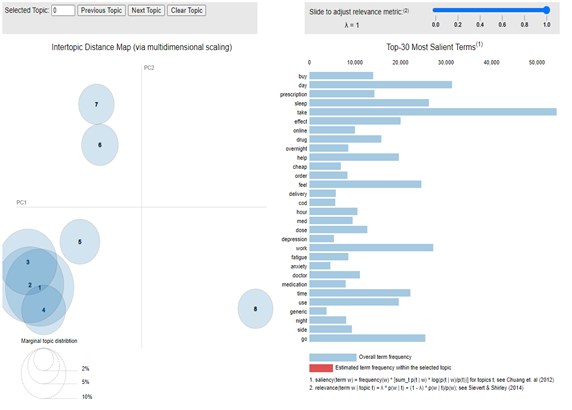

Supplement: Multimedia Appendix 2 [file jmir_v26i1e54321_app2.png]

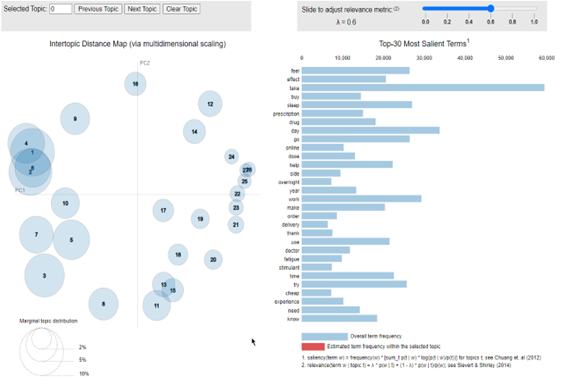

Supplement: Multimedia Appendix 3 [file jmir_v26i1e54321_app3.png]

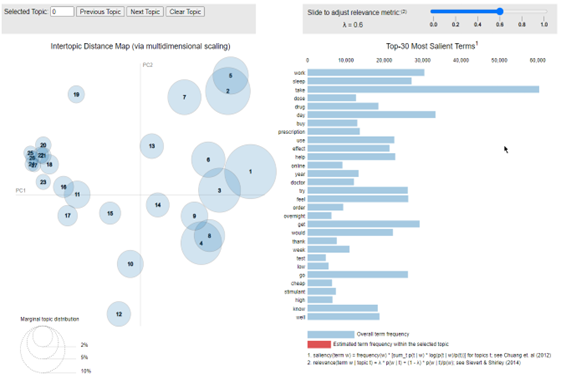

Supplement: Multimedia Appendix 4 [file jmir_v26i1e54321_app4.png]
