# Supplementary material for: Combining Topic Modeling, Sentiment Analysis, and Corpus Linguistics to Analyze Unstructured Web-Based Patient Experience Data: Case Study of Modafinil Experiences
Source: J Med Internet Res. 2024 Dec 11;26:e54321. doi: 10.2196/54321 (PMC11669883; doi:10.2196/54321)
Supplement: Multimedia Appendix 5 [file jmir_v26i1e54321_app5.docx]

Table 4: Comparison of sklearn LDA and NMF topics (n=27)

| sklearn LDA 27 topics | | | | |  | sklearn NMF 27 topics | | | | |
| --- | --- | --- | --- | --- | --- | --- | --- | --- | --- | --- |
| Topic No | No Docs | % of Docs | Top 10 topic words | Theme |  | Topic No | No Docs | % of Docs | Top 10 topic words | Theme |
| 15 | 13774 | 20.09 | fatigue take day sleep get work help like taking feel |  |  | 0 | 11361 | 16.57 | would know think good one also really could much something |  |
| 26 | 10685 | 15.59 | like would take caffeine also noopept get day effects taking |  |  | 26 | 3731 | 5.44 | time first took got started years ago today last back |  |
| 11 | 10558 | 15.40 | take day feel taking 200mg like effects dose caffeine 100mg | Dosage |  | 1 | 3573 | 5.21 | sleep night apnea study hours awake asleep wake narcolepsy cpap |  |
| 2 | 7421 | 10.82 | order generic modalert uk buy get online brand prescription com | Acquisition |  | 2 | 3010 | 4.39 | fatigue ms helps amantadine chronic neuro pain helped prescribed years |  |
| 24 | 7195 | 10.49 | sleep narcolepsy doctor get apnea know fatigue help work would |  |  | 13 | 2731 | 3.98 | generic modalert brand name sun order modvigil pills pharma price |  |
| 13 | 5994 | 8.74 | depression wellbutrin adderall meds take anxiety help adhd like bipolar |  |  | 7 | 2559 | 3.73 | effects side effect negative term headaches experience long bad experienced | SideEffects |
| 8 | 4658 | 6.79 | dopamine adrafinil effects would like amphetamine liver drug effect drugs |  |  | 20 | 2535 | 3.70 | caffeine coffee theanine drink moda noopept nicotine cup tea energy |  |
| 23 | 4471 | 6.52 | like get people sleep time would think drugs take really |  |  | 16 | 2535 | 3.70 | anxiety depression meds wellbutrin adhd bipolar add mood lamictal treatment |  |
| 9 | 554 | 0.81 | fatigue anyone take helps tried help helped rs4680 works work |  |  | 9 | 2449 | 3.57 | insurance cover pay company doctor month expensive cost covered generic | Acquisition |
| 25 | 447 | 0.65 | buy online prescription overnight cod delivery cheap order without http | Acquisition |  | 6 | 2383 | 3.48 | dose 200mg 100mg 50mg morning mg dosage low doses half | Dosage |
| 12 | 424 | 0.62 | www http com gov nlm ncbi html https nih greater | Info |  | 23 | 2341 | 3.41 | http www com html https org uk nlm ncbi reddit | Info |
| 10 | 298 | 0.43 | insurance entry div commenttext itemprop content class cover generic post |  |  | 5 | 2316 | 3.38 | adderall vyvanse vs xr adhd amphetamine dexedrine add ir mg | Other Int |
| 21 | 281 | 0.41 | vitamin water oil bulletproof diet coffee supplements vit magnesium drink |  |  | 12 | 2115 | 3.08 | feel like makes tired feeling awake better really felt normal | Effect |
| 5 | 245 | 0.36 | birth narcolepsy drug control world smart safe decision concluded creatively |  |  | 10 | 2048 | 2.99 | anyone tried else experience know wondering thanks ever taken experiences | Question |
| 20 | 217 | 0.32 | nardil heart parnate blood blunting pressure anxiety quote irritable emotional |  |  | 11 | 1964 | 2.86 | adrafinil liver prodrug 300mg vs metabolized noopept 600mg legal powder |  |
| 14 | 209 | 0.30 | smart bbc limitless nightmare drugs documentary movie nzt semax journalist | Media |  | 24 | 1942 | 2.83 | ritalin adhd concerta tried stimulants methylphenidate stimulant amphetamines vs add | Other Int |
| 6 | 202 | 0.29 | nsi 189 tapatalk chemically ltp sent tinnitus iphone induced saphris |  |  | 18 | 1907 | 2.78 | use days tolerance week daily using long term build phenylpiracetam | Dosage |
| 19 | 161 | 0.23 | mdma ly master monk asshole mode oral smarter testosterone gabapentin |  |  | 8 | 1904 | 2.78 | day every awake night twice per hours next stay one | Dosage |
| 22 | 152 | 0.22 | sjs rash skin reaction allergic johnson syndrome stevens throat itchy | SideEffects |  | 4 | 1884 | 2.75 | take helps morning also need days sometimes everyday wake needed |  |
| 16 | 119 | 0.17 | hair loss regrow sores contraception tbi strike nuvagil msm expired |  |  | 14 | 1861 | 2.71 | drug narcolepsy people smart prescribed medication drugs safe world prescription |  |
| 17 | 101 | 0.15 | trashy addicts phentermine favourite assistance thread claritin xd dexamphetamine nootriment |  |  | 17 | 1826 | 2.66 | taking started stop stopped week daily months weeks headaches years | Dosage |
| 3 | 89 | 0.13 | banned esports tournaments italy ban psychoactive uk bill doping microdoses | Legal |  | 3 | 1805 | 2.63 | buy online prescription overnight cod delivery order cheap without pharmacy | Acquisition |
| 1 | 85 | 0.12 | ireland geoff rx_rex custom hillary swd aching disease reporter gotmilk |  |  | 15 | 1663 | 2.43 | work shift disorder working well job home shifts worked full |  |
| 0 | 69 | 0.10 | stacks er visit fear finasteride vs trying rc caffeine wat |  |  | 19 | 1621 | 2.36 | help need please awake energy stay focus meds pain tried |  |
| 18 | 55 | 0.08 | pregnancy jaw teeth authoritative breastfeeding clenching nursing guide grinding ltheanine |  |  | 21 | 1567 | 2.29 | get done need go able bed back prescription script hard |  |
| 7 | 47 | 0.07 | netherlands kg cons pros hype burnout xxx shortage procrastination dpd |  |  | 25 | 1521 | 2.22 | try give see want might maybe going could thanks next |  |
| 4 | 40 | 0.06 | pots tension intas forehead hcg agomelatine muscle reminder friendly ppap |  |  | 22 | 1372 | 2.00 | works well great better worked hope best wonders find tried | Effect |
| 28 | 8 | 0.01 | None |  |  | 28 | 35 | 0.05 | None |  |
|  |  |  |  |  |  |  |  |  |  |  |
| Total | 68559 | 100 |  |  |  | Total | 68559 | 100 |  |  |
