# Supplementary material for: Combining Topic Modeling, Sentiment Analysis, and Corpus Linguistics to Analyze Unstructured Web-Based Patient Experience Data: Case Study of Modafinil Experiences
Source: J Med Internet Res. 2024 Dec 11;26:e54321. doi: 10.2196/54321 (PMC11669883; doi:10.2196/54321)
Supplement: Multimedia Appendix 6 [file jmir_v26i1e54321_app6.docx]

| **Word sketch, collocates and frequencies of ‘feel’ from corpus** | | |
| --- | --- | --- |
| **Gramrel** | **Collocate** | **Freq** |
| modifiers of feel |  | 6842 |
|  | still | 589 |
|  | awake | 227 |
|  | never | 242 |
|  | absolutely | 46 |
|  | kinda | 36 |
|  | alone | 25 |
|  | constantly | 22 |
|  | ill | 20 |
|  | wide | 14 |
|  | emotionally | 13 |
|  | sorry | 12 |
|  | fully | 12 |
|  | strongly | 9 |
|  | tight | 7 |
|  | afterwards | 7 |
|  | overly | 7 |
|  | guilty | 6 |
|  | close | 6 |
| objects of feel |  | 4354 |
|  | bit | 252 |
|  | effect | 477 |
|  | way | 220 |
|  | need | 134 |
|  | nothing | 167 |
|  | fine | 101 |
|  | difference | 91 |
|  | pain | 64 |
|  | alert | 54 |
|  | today | 61 |
|  | kind | 54 |
|  | urge | 24 |
|  | energy | 31 |
|  | emotion | 22 |
|  | day | 46 |
|  | alot | 21 |
|  | crash | 19 |
|  | sense | 21 |
|  | spacey | 17 |
|  | driving | 17 |
|  | euphoria | 15 |
|  | crappy | 14 |
|  | sensation | 12 |
|  | brain | 13 |
|  | buzz | 11 |
|  | rush | 11 |
|  | hyper | 11 |
|  | opposite | 10 |
|  | pressure | 10 |
|  | pleasure | 9 |
|  | hunger | 8 |
|  | exhaustion | 7 |
|  | crap | 7 |
|  | etc. | 7 |
|  | affect | 7 |
|  | while | 7 |
|  | discomfort | 6 |
|  | decrease | 6 |
|  | light | 6 |
|  | level | 7 |
|  | doctor | 6 |
| subjects of feel |  | 2032 |
|  | i | 634 |
|  | dont | 37 |
|  | body | 45 |
|  | day | 38 |
|  | head | 22 |
|  | eye | 22 |
|  | people | 72 |
|  | heart | 17 |
|  | Modalert | 16 |
|  | morning | 13 |
|  | stomach | 11 |
|  | everything | 13 |
|  | life | 13 |
|  | eyelid | 8 |
|  | chest | 8 |
|  | user | 8 |
|  | question | 8 |
|  | im | 8 |
|  | throat | 6 |
|  | havent | 6 |
| feel and/or ... |  | 150 |
|  | yawn | 8 |
|  | think | 9 |
|  | look | 6 |
|  | sleep | 10 |
| prepositional phrases |  | 2163 |
|  | "%w" like ... | 1590 |
|  | "%w" in ... | 98 |
|  | "%w" on ... | 69 |
|  | "%w" of ... | 47 |
|  | "%w" for ... | 43 |
|  | "%w" if ... | 34 |
|  | "%w" about ... | 33 |
|  | "%w" with ... | 31 |
|  | "%w" at ... | 25 |
|  | "%w" after ... | 23 |
|  | "%w" though ... | 22 |
|  | "%w" to ... | 21 |
|  | "%w" from ... | 21 |
|  | "%w" during ... | 20 |
|  | "%w" as ... | 18 |
|  | "%w" before ... | 8 |
|  | "%w" over ... | 6 |
| particles after feel |  | 74 |
|  | down | 13 |
|  | off | 15 |
|  | out | 21 |
|  | up | 23 |
| particles after feel with object |  | 19 |
|  | out | 6 |
|  | up | 7 |
| pronominal objects of feel |  | 689 |
|  | myself | 35 |
|  | it | 540 |
|  | them | 24 |
|  | you | 74 |
| pronominal subjects of feel |  | 12026 |
|  | me | 1467 |
|  | I | 7620 |
|  | you | 1641 |
|  | it | 839 |
|  | he | 129 |
|  | they | 129 |
|  | she | 61 |
|  | we | 39 |
|  | him | 25 |
|  | them | 24 |
|  | myself | 14 |
|  | one | 14 |
|  | us | 11 |
|  | her | 9 |
| wh-words following feel |  | 179 |
|  | when | 125 |
|  | that | 14 |
|  | which | 6 |
|  | what | 20 |
|  | how | 8 |
| infinitive objects of feel |  | 37 |
|  | be | 13 |
| -ing objects of feel |  | 81 |
|  | amaze | 7 |
|  | fuck | 6 |
|  | take | 12 |
| complements of feel |  | 289 |
|  | different | 19 |
|  | weird | 7 |
|  | full | 6 |
|  | alert | 7 |
| adjectives after feel |  | 5342 |
|  | tired | 492 |
|  | good | 782 |
|  | free | 223 |
|  | normal | 170 |
|  | awake | 100 |
|  | comfortable | 81 |
|  | different | 93 |
|  | weird | 70 |
|  | terrible | 53 |
|  | guilty | 51 |
|  | amazing | 49 |
|  | confident | 30 |
|  | happy | 32 |
|  | shitty | 27 |
|  | alive | 22 |
|  | heavy | 21 |
|  | fine | 23 |
|  | hopeless | 17 |
|  | sluggish | 17 |
|  | euphoric | 16 |
|  | full | 16 |
|  | cold | 15 |
|  | helpless | 12 |
|  | suicidal | 12 |
|  | human | 11 |
|  | dirty | 11 |
|  | stable | 10 |
|  | flat | 9 |
|  | itchy | 8 |
|  | ready | 8 |
|  | fresh | 7 |
|  | natural | 7 |
|  | super | 7 |
|  | slow | 7 |
|  | most | 7 |
|  | fake | 6 |
|  | everyday | 6 |
|  | useless | 6 |
| feel like ... |  | 1590 |
|  | shit | 101 |
|  | i | 99 |
|  | zombie | 68 |
|  | person | 68 |
|  | self | 23 |
|  | stimulant | 18 |
|  | heart | 15 |
|  | im | 14 |
|  | superman | 11 |
|  | head | 11 |
|  | human | 11 |
